# Ectopic intrathyroidal thymic tissue in an adult patient: a case report and review of the literature

**DOI:** 10.1186/s13256-022-03434-3

**Published:** 2022-05-25

**Authors:** Sadegh Moradian, Sina Delazar, Farzad Yazdani, Maryam Mohammadzadeh

**Affiliations:** 1 Department of Radiology, Tehran University of Medical Sciences, Tehran, Iran; 2 Department of pathology, Amiralam Hospital, Tehran University of Medical Sciences, Tehran, Iran; 3 Division of Neuroradiology and Head & Neck Imaging, Department of Radiology, Tehran University of Medical Sciences, Tehran, Iran

**Keywords:** Ectopic thymus, Intrathyroidal lesion, Adult, Ultrasonography, Case report

## Abstract

**Background:**

Ectopic intrathyroidal thymic tissue is a rare diagnosis, specifically in adults. Such ectopic tissue is usually misdiagnosed as benign or malignant thyroid lesions and is mainly investigated by ultrasonography and pathologic examination.

**Case presentation:**

We present the case of an adult 31-year-old Persian female patient with a cervical mass and no other significant medical history. The lesion had hypo- to isoechoic features on sonographic imaging, and needle aspiration examination revealed lymphoid cells suspicious of lymphoid malignancies. However, pathologic examination after resection of the lesion showed normal thymic tissue.

**Conclusions:**

The rare entity of ectopic thymic tissue within the thyroid gland in adult patients requires meticulous examination by clinicians, radiologists, and pathologists to diagnose the condition with high accuracy and plan appropriate management.

## Background

Ectopic intrathyroidal thymic tissue is a rare condition that is frequently seen in pediatric patients but extremely rarely in adults, being found incidentally on cervical imaging or usually misdiagnosed as benign or malignant thyroid lesions such as nodules or malignancies [1, 2]. This rare condition is often seen during the prepubertal period when the thymus undergoes its fastest growth stage, and the massive thymus within the thyroid becomes apparent [1, 3]. Later, at adult ages, the thymus goes through a process named involution, and the adipose tissue replaces the thymic tissue in adults. Therefore, diagnosis of persistent thymic tissue in adults within or outside the thyroid is exceedingly rare [2, 3]. Ultrasonography (US) has been reported to be the best modality for initial investigation of such lesions, as it is noninvasive and easily available [4, 5]. We report herein the case of an adult patient diagnosed finally with ectopic intrathyroidal thymic tissue and review relevant previous literature on the condition in adults, to provide evidence for this rare pathology for better management of probable cases in the future.

## Case presentation

We present the case of a 31-year-old Persian female patient with a history of a mass in the left side of the neck for more than a year before referral to the clinic. The patient was gravid 2 with two children, living in the city center and working as a high-school teacher. The patient had no history of smoking or alcohol or drug use. The patient had no history of hyperthyroidism or hypothyroidism, or constitutional symptoms. History of diabetes mellitus, hypertension, and hyperlipidemia was also negative in this case. Also, the patient had no significant past medical history nor family history of ectopic cervical masses or thyroid gland lesions. The patient was using no medications when referred to our medical center.

On physical examination at admission, the vital signs were normal with blood pressure of 120/80 mmHg, pulse rate of 85 per minute, and temperature of 37 °C. The primary evaluation of all 12 cranial nerves was normal. On neck examination, a palpable firm mass without tenderness was present in the left lobe of the thyroid. On auscultation of thyroid, no bruit was detected. Thyroid function tests were normal (thyroid stimulating hormone 1.5 mIU/L, free T4 = 1.1 ng/dL, T3 = 150 ng/dL), and the patient was euthyroid at presentation. Also, all other laboratory tests were normal (WBC = 8000/mL, hemoglobin = 12 g/dL, hematocrit = 40%, platelets = 250,000/mL, alanine aminotransferase = 20 units/L, aspartate aminotransferase = 10 units/L, alkaline phosphatase = 60 IU/L, urine analysis = normal, urine culture = no growth).

**Fig. 1:**
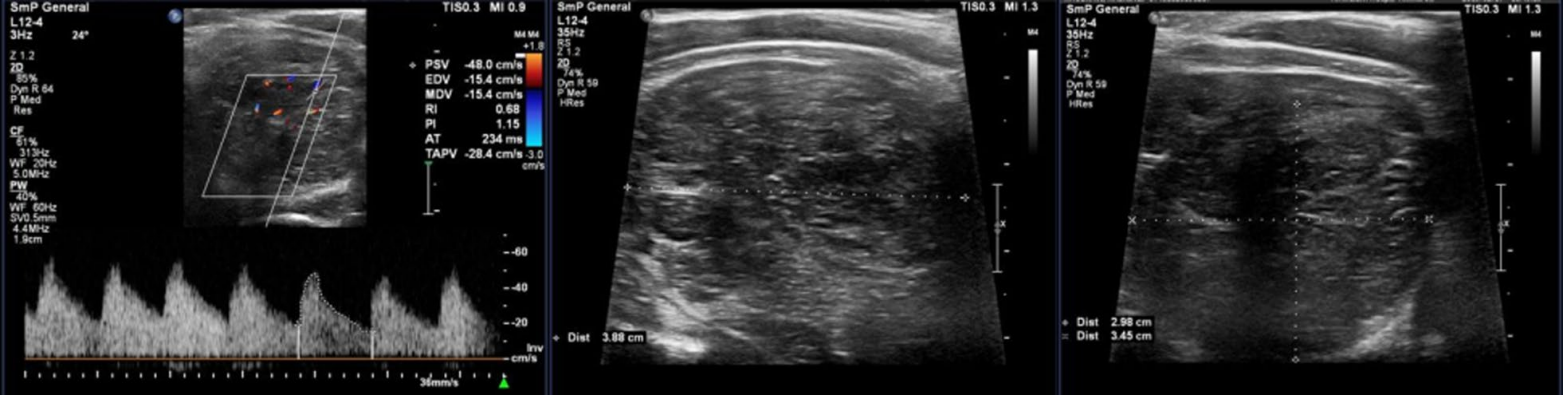
Ultrasonography evaluation of the suspected lesion

In US of neck and thyroid, a hypo- to isoechoic nodule with dimensions of 2.98 × 3.45 × 3.88 cm^3^ with internal striation and cystic areas (less than 50% of mass) was detected in the lower pole of the left lobe of the thyroid gland. The lesion had no micro- or macrocalcification in the examination. There was no evidence of cervical lymphadenopathy in the US investigation (Fig. 1). Fine needle aspiration (FNA) of the lesion was done, and the microscopic examination of prepared slides showed many, varied size lymphoid cells containing some atypical large lymphoid cells with high nuclear-to-cytoplasmic (N/C) ratio and prominent nucleoli. The cytopathology of the specimen diagnosed tissue suspicious for lymphoproliferative disorder, and excisional biopsy of the mass was recommended. The patient underwent surgical excision of the lesion through total thyroidectomy, and the postoperative pathology examination revealed thymic tissue. Six months after surgery, the patient was referred for reevaluation; all physical examination and laboratory tests were rechecked and found to be normal.

**Fig. 2:**
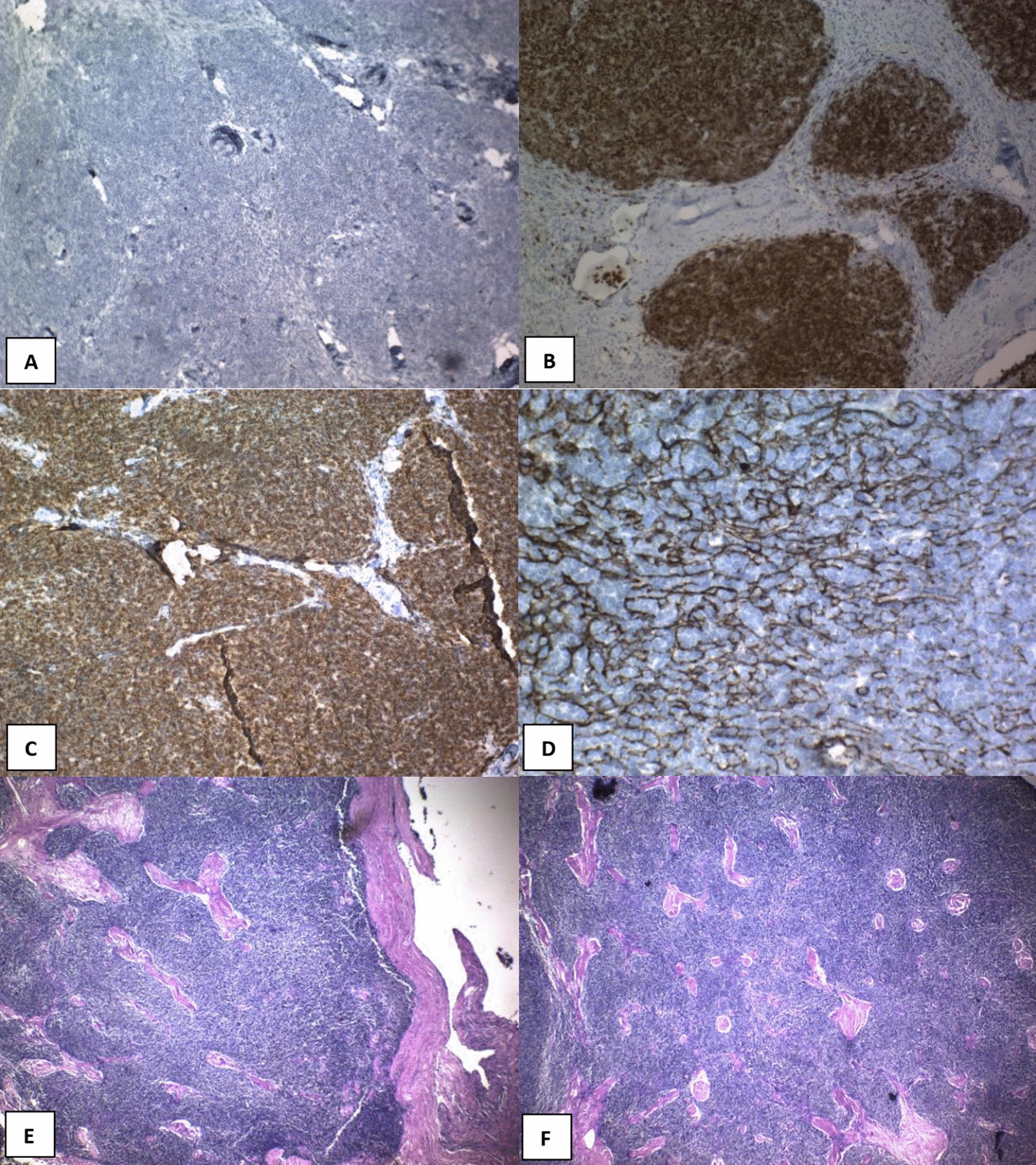

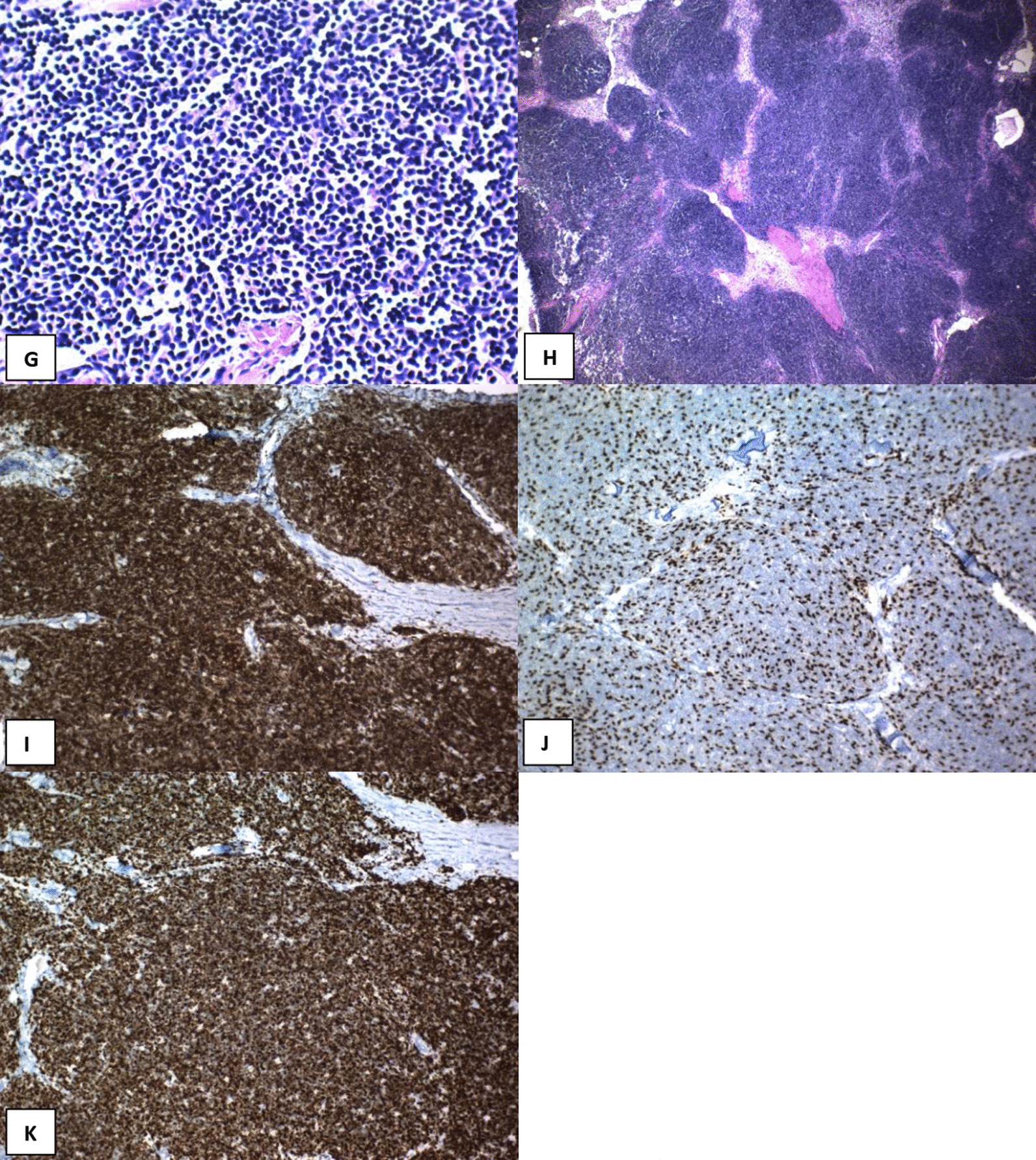
Microscopy: H&E sections (**E**–**H**) show an encapsulated mass with a thick fibrous capsule and intersecting fibrous bands separating it into lobulated architecture with cellular lobules resembling the normal thymic cortex composed of scattered bland thymic epithelial cells in a dense background of small lymphocytes (Immature T cells). Immunohistochemistry: The scattered epithelial tumoral cells show immunoreactivity for CKAE1/AE3 (**D**) and P63 (**J**). Background lymphoid cells show diffuse immunoreactivity for LCA (Leukocyte common antigen), CD3 (**K**), CD43 (**B**), and CD99 (**C**) but are negative for CD20 (**A**). The proliferation index (KI67) (**I**) is very high in lymphoid cells

On microscopic assessment of the excised lesion, hematoxylin and eosin (H&E)-stained sections showed an encapsulated mass with thick fibrous capsule and intersecting fibrous bands separating it into lobulated architecture with cellular lobules resembling normal thymic cortex, which were composed of scattered bland thymic epithelial cells in a dense background of small lymphocytes (immature T cells). On immunohistochemistry (IHC) evaluation, the scattered epithelial tumoral cells showed immunoreactivity for cytokeratin AE1/AE3 (CK AE1/AE3) and P63. Background lymphoid cells showed diffuse immunoreactivity for leukocyte common antigen (LCA), CD3, CD43, and CD99; however, they were negative for CD20. The Ki-67 proliferation index was very high in lymphoid cells (Fig. 2).

## Discussion

We present the case of an adult female patient without any previous medical history and with a firm mass on neck examination besides normal thyroid function and laboratory tests, who was diagnosed with ectopic intrathyroidal thymic tissue, which has been rarely reported, in a few case reports before. The clinical and pathologic features presented by this case provide evidence for the management of similar cases with this rare diagnosis. A systematic search found four similar papers previously reporting ectopic thymus in thyroid in adult patients [2, 4, 6, 7]. It is interesting that all of the reported cases were consistent in the female sex of the patients.

Moon *et al.* reported a 31-year-old female patient with left ectopic intrathyroidal thymic tissue and a right papillary thyroid carcinoma (PTC) that first presented with enlargement of the right lobe of the thyroid with low serum thyrotropin and elevated free thyroxin in laboratory tests. Their investigation with US found a hypoechoic mass in the right lobe later, confirmed as PTC, but nothing in the left lobe. Further examination by computed tomography (CT) revealed a small hypoattenuating nodule in the left lobe in the precontrast phase and no enhancement in the postcontrast phase. The patient later underwent total thyroidectomy, and histological examination of the left lobe found mature adipose tissue, lymphoid tissue, and Hassall’s corpuscles, compatible with thymic tissue [2].

Kim *et al.* reported a 29-year-old woman with history of right thyroidectomy due to PTC 3 years before her referral and a right neck mass. The initial examination with US showed a well-defined hyperechoic nodule in the left lobe of the thyroid. The patient underwent total thyroidectomy with right neck dissection. On gross examination, a well-defined pale-yellow nodule of 0.9 cm was seen in the left thyroid lobe, which on microscopic examination, mature adipose tissue, ectopic thymic tissue with Hassall’s corpuscles, and intrathyroidal parathyroid tissue was found [6].

O’Connor *et al.* reported a 23-year-old female patient with Grave’s disease refractory to medical management and a history of clinical hyperthyroidism, exophthalmos, and asthma. Cervical US showed a hypervascular and enlarged thyroid, and the gland had diffuse homogeneous radioiodine uptake on nuclear imaging. The patient underwent total thyroidectomy and right inferior parathyroidectomy, which was enlarged during the surgical exploration. Surgical pathology and histopathology examination of the excised tissues showed diffuse hyperplasia of thyroid tissue with colloid involution consistent with the treated Grave’s condition and focal ectopic intrathyroidal thymic tissue and some chondroid metaplasia [4].

Velimezis *et al.* reported a 26-year-old woman with hyperthyroidism controlled with levothyroxine and a heterogeneous 1.15-cm nodule containing microcalcifications in the right thyroid lobe, suggesting the presence of malignancy on the cytology examination. The patient underwent total thyroidectomy, and postoperative histopathology examination found multifocal PTC associated with ectopic intrathyroidal thymic and parathyroid tissues in the right thyroid lobe. The patient had no complications in the 2-year follow-up period after the surgery [7].

Ectopic intrathyroidal thymic tissue has been studied in more detail in pediatric patients because it is more prevalent at lower ages. Ectopic thymic tissue results from deviation in the normal migration path of the thymus, which originates from the third pharyngeal pouches and moves medially and caudally to locate at the superior mediastinum by the eighth week of embryogenesis [2, 4, 5]. Being adjacent to the origin of inferior parathyroid glands and developing in a path through the thyroid, the presence of ectopic tissues of these three organs near each other is possible, as presented herein and the reviewed literature [1, 7]. A large-scale study of 375 cases of pediatric patients with ectopic intrathyroidal thymic tissue proposed that the typical findings of this condition on US are irregular, triangular, or polygonal hypoechoic or hyperechoic areas with punctate, granular, and linear echogenic foci inside the lesions [1]. A systematic review on ectopic intrathyroidal thymic tissue in children proposed that the cytology appearance of this lesion is benign-appearing lymphocytes, often with minimal or no epithelial cell components [8]. Also, this systematic review found the classic appearance of a hypoechoic mass with sharp margins and multiple focal internal echogenicities without shadows, just like normal thymic tissue in the US exploration of the lesion [8].

Critical differential diagnoses of an ectopic intrathyroidal mass full of lymphocytes are acute lymphoid leukemia, thymic carcinoma, carcinoma showing thymus-like differentiation (CASTLE), spindle cell tumors with thymus-like differentiation, and thymoma [8]. Thymic tissue present within the thyroid gland can go through hyperplastic and metaplastic changes and result in benign and malignant lesions such as intrathyroidal epithelial thymoma (ITET) and CASTLE [2, 6]. Despite the rare prevalence of ectopic presence of thymic tissue in the thyroid gland, transformation to malignancies in this ectopic tissue should be kept in mind as series of patients with tumors such as ITET/CASTLE have been reported, and the appropriate management of the condition requires adequate awareness of it [9].

As this condition is rare, overall and exceptionally in adults, the management of the lesion is subject to great doubt and remains unknown. However, all the reviewed cases, similar to the current reported case, underwent total thyroidectomy with no need for further intervention except follow-up and close monitoring of surgical complications. A previously mentioned study proposed an algorithm for diagnosing and evaluating ectopic intrathyroidal thymic tissue through a comprehensive systematic review of available literature on pediatric patients [8]. According to this algorithm, if the lesion has definitive US features of thymic tissue, no further evaluation or follow-up is needed. Otherwise, if the lesion is smaller than 10 mm, deferring surgery and monitoring with serial US is suggested. For lesions ≥ 10 mm, US-guided FNA is suggested. Next, running flow cytometry using CD4 and CD8 markers is suggested to differentiate the lesion as mature thymic tissue from T lymphoblastic leukemia and thymic carcinoma [8]. However, one should bear in mind that this algorithm is proposed based on evidence from pediatric patients, and applying it to adults should be done with caution.

## Conclusion

The rare condition of ectopic thymic tissue within the thyroid gland, without symptoms, continuing to adult age, requires refined clinical and paraclinical awareness and attention to manage the patient with the least unnecessary interventions and surgical procedures. Also, clinicians with crucial roles in this path such as radiologists and pathologists must pay special attention to this rare manifestation of a congenital remnant in adulthood. More investigations and evidence are needed to clarify the proper management of these patients, as clinicians face a dilemma regarding conducting unnecessary surgery or accepting the risk of neoplasm formation from the heterotopic tissue of the thymus within the thyroid gland.

## Data Availability

Not applicable.
